# Deduction with uncertain conditionals (revised & simplified, with examples)

**DOI:** 10.1016/j.heliyon.2021.e08328

**Published:** 2021-11-05

**Authors:** Philip G. Calabrese

**Affiliations:** Data Synthesis, 2919 Luna Avenue, San Diego, CA 92117, USA

**Keywords:** Conditional, Deduction, Quasi-conjunction, Implication, Inference, Uncertainty, Probability

## Abstract

Section 1 of this paper provides an introduction to this new “algebra of conditionals”, addresses various plausibility tests for such an algebra, provides a Venn diagram disproving a supposed counter-example, and answers various other objections raised in the literature about the efficacy of this algebraic extension of logic and conditional probability. Section 2 greatly simplifies the calculation of the implications of a set of conditional propositions or conditional events. These results depend on defining a deductive relation for conditionals (actually two have been found) with the property that the conjunction of two conditionals implies each of its components. That seemingly innocuous property assures that the deductively closed set implied by a finite set of n conditionals with respect to the deductive relation is implied by the single conditional formed by conjoining all n of them. The results are illustrated by solving several examples of deduction with several uncertain conditionals.

## 1 Introduction

The general topic of conditional events and conditional probability continues to be an active area of research, [1, 2, 6]. The main purpose of this paper is to greatly simplify my previous expositions of deduction with partially true conditionals^1^. This simplification is due to the discovery of two new (at least to me) deductive relations on conditional events (and conditional propositions) that greatly simplify the problem of computing the implications of a set of two or more possibly uncertain conditionals.

This problem arose when two Boolean conditionals (a given b) & (c given d) were “quasi-conjoined” and expressed as a single Boolean conditional (ab or cd) given (b or d), which (depending on the implication relation adopted) might not imply one or both of its components! It became necessary in theory to form all finite conjunctions of the assumed conditionals in order to compute their combined implications. Due to complexity, this was impractical even for a computer. This paper will eliminate that issue and also answer some objections to the idea of treating conditionals whose components are Boolean propositions or probabilistic events as trivalent ordered pairs with operations that extend those of Boolean algebra.

### 1.1 The necessity of conditionals

It is commonly thought that explicitly conditional statements such as “When it rains an umbrella is useful” are somehow different from (non-explicitly conditional) statements formed by purely Boolean combinations of “properties”, “propositions” or probabilistic “events”. However, it is impossible to formulate a completely unconditional statement! They all have an assumed context and a qualification within that context. “Redness” entails the context of “color”; Truth entails the potential context of its absence. Any assertion requires a conceptual framework in which to make it.

It becomes clear that information should really come as an ordered pair of statements, (a|b) --- “a given b”, “a in the context of b”, “a if b” or “if b then a”. The first two of these constructions are not necessarily absolute implications but merely a qualification “a” in the context of a condition “b”. Usually, the last two of these constructions mean “b necessarily implies a”, although this distinction has not always been made.

### 1.2 The logic-probability, intension-extension connection

By information I mean the indicative conditional propositions of logic and their associated conditional events of probability theory. Each proposition q has an associated subset of models (worlds, examples) in which it is unambiguously true, and the probability of q is defined to be the probability of those models in which it is true. Thus, propositions can be represented as measurable indicator functions defined on the universe of models^2^.

### 1.3 Definition of the conditional closure

Let (B|B) denote the collection of all order pairs (a|b), where a, b are Boolean propositions or probabilistic events. (B|B) will be called the *Conditional Closure* of the Boolean algebra B, and its members will be called *conditional propositions* (or *conditional events*, when viewed as subsets of models).

An algebraic extension algebra of Boolean logic and probability logic must avoid the 2-valued logical simplification of including in the truth of a conditional statement or implication “a if b” all instances of the falsity of the premise b. Translating “a if b” as “either a or not b”, the so-called material conditional [24], takes the 2-valued position that if a conditional is “not false”, then it must be true; and it is “false” only when “a” is false and b is true. Thus “a if b” has been classified as true when either its premise b is false or its qualification a, is true. A false premise supposedly guarantees the truth of any conclusion!? However, a valid proof by contradiction must be based on true premises except the one being conjectured.

While 2-valued conditionals and implications adequately serve absolute (2-valued) logical thinking, they severely distort conditional probabilities by not adequately distinguishing between instances of a false premise from those for which both the components of the conditional are true. This is why it is necessary to have 3 truth categories: “true”, “false” and “inapplicable” (undefined).

D. Lewis [7] early showed that (a|b) could not be assigned the conditional probability P(a|b) and also be a member of the original Boolean algebra containing a and b because then, using standard conditional probability rules, P(a|b) = P((a|b) ∧ (a ∨ a′)) = P((a|b) ∧ a) + P((a|b) ∧ a′) = P((a|b) | a)P(a) + P((a|b) | a′)P(a′) = P(a|ba)P(a) + P(a|ba′)P(a′) = (1)P(a) + (0)P(a′) = P(a) no matter what (except for trivial cases) the propositions a and b!

Although no logical object (a|b) is explicitly included in the standard formulation of either Boolean algebra or Kolmogorov probability theory, there is an *extension algebra* [3] of ordered pairs that does in fact include all these algebraic objects (a|b), and assigns them (conditional) probabilities consistent with both Boolean logic and probability theory.

This partially distributive extension algebra of ordered pairs of propositions or events allows the disjunction or conjunction of two or more conditionals with different premises to be combined into as a single conditional event with a well-defined conditional probability.

### 1.4 Definition of equivalence^3^

Two of these pairs (a|b) and (c|d) are considered equivalent (=) in case their conditions (b & d) are equivalent, and also their identified qualifications or conclusions (a & c) are equivalent when conjoined with their equivalent conditions. That is, (a|b) = (c|d) if and only if b = d and ab = cd, where juxtaposition means Boolean conjunction. Note that (a|b) = (ab|b).

### 1.5 Three-valuedness

Such a pair (a|b), “a given b”, appears at first to have 4 truth states: (True|True), (False|True), (True|False) and (False|False). The first two reduce to “True” and “False” respectively when the given condition is true. However, according to the above definition the last two states are equivalent because their conditions are both false (and so equivalent), and conjoined with those false premises their identified qualifications are also false and so equivalent. In this last state the conditional is said to be “inapplicable” or undefined. Notice, in this 3-valued system, “not false” is no longer the same as “true”. It means “true or inapplicable”.

While it is often possible in symbolic logic to maintain a constant context and therefore avoid the complexities of changing conditions, the inherent inadequacy of such an approach is clear when propositions that are only partially true enter the picture as in probability theory. Such propositions can be true in some models (instances) but false in others. At that point the inability to classify a proposition in any other way besides “always true” or “not always true” (false) becomes inhibiting.

After all, inapplicability is all around us in every statement or proposition. Since every statement has a premise, it does not apply in situations that falsify that premise. Thus, for any instance or outcome (conditional) propositions have one of three mutually exclusive truth-values: true, false and inapplicable (or undefined).

### 1.6 Operating on conditionals

Any extension of Boolean operations to two conditionals (a|b) and (c|d) must of course reduce to ordinary Boolean operations of conjunction (∧; and), disjunction (∨; or) and negation (′; not) whenever b = d, that is, whenever the conditions are equivalent. This is true for the following extended operations on conditionals^4^ having possibly non-equivalent conditions.

1) (a|b) ∨ (c|d) = (ab ∨ cd |b ∨ d)
2) (a|b) ∧ (c|d) = (abd′ ∨ cdb′ ∨ abcd |b ∨ d) = ((a ∨ b′) (c ∨ d′) |b ∨ d)
3) (a|b)′ = (a′|b)

In the conditional closure, a formally unconditioned (universally conditioned) proposition or event, a, is identified with the conditional (a|1), where 1 is the unique “always true” logical unit (b ∨ b′) or in probability theory the universal event Ω having probability 1. All propositions or events a, b that make up a conditional (a|b) are assumed to be members of a fixed universe of all possibilities.

These operations on conditionals were chosen to be fully compatible with the well-established “conditional probability function” P(a|b) of “proposition a given b” defined to be the ratio P(a ∧ b)/P(b).

The disjunction of two conditionals “(a given b) or (c given d)” expands the context to (b ∨ d) and qualifies this context with the proposition ab ∨ cd, which is the disjunction of the applicable qualifications of the component conditionals.

The conjunction of these same two conditionals equally expands the context but further qualifies that context to the truth (ab) of the first conditional conjoined with the inapplicability (d′) of the second conditional plus (or) the truth of the second conditional conjoined with the inapplicability of the first conditional plus the conjunction (abcd) of the truths of both conditionals.

To show this conjunction of the two conditionals is also the conditional ((a ∨ b′) (c ∨ d′) |b ∨ d) one need only expand (ab ∨ b′) (cd ∨ d′) and condition the result by (b ∨ d).

### 1.7 Non-monotonic operations. A die example

As noted above, when two conditionals with different premises are conjoined or disjoined their context is expanded to the disjunction of their premises. This can result is an object whose probability is “non-monotonic” with respect to its component conditional propositions. The conjunction of two conditionals can have a larger probability than both of its components! A disjunction can be less probable than each of its components.

For instance, consider the experiment of rolling a familiar single 6-sided die, and suppose I bet “if the roll is an even number, it will be a 2 or 4, and if the roll is 5 or 6, it will be 5.” The compound conditional ((2 or 4 | Even) ∧ (5 | 5 or 6)) equals the single conditional event (2, 4 or 5 given “Even or 5 or 6”) and it has a well-defined conditional probability. The conditional event is (2, 4, 5 given the roll is even or 5) and so it has a conditional probability of 3/4.

Note however that the conditional probability of (2 or 4 | Even) is 2/3. Conjoining this with (5 | 5 or 6), which has conditional probability 1/2, yields the compound conditional (2, 4, or 5 given the roll is even or 5), which has probability ¾, which is larger than both 2/3 and 1/2 despite the conjoining of the two conditionals! But this is simply the way we linguistically and probabilistically combine conditionals. E. Adams called these operations “quasi-” because of their non-monotonic properties.

Nevertheless, quasi-conjunction is the only operation that is consistent with conditional probability and that also affirms “proof by cases”. That is, a proposition C can be proved true by showing “C is true if D is true, and C is true if D is false”, which can be expressed as (C|D) ∧ (C|D′), which by quasi-conjunction equals (C |D ∨ D′), which is “C given D is true or not true” = C.

### 1.8 Reversion to the material conditional

Notice that according to the operations, when a conditional (c|d) is *conjoined* with an unconditioned (i.e. universe conditioned) proposition b the result is b(c ∨ d′); it reverts to its material conditional because b ∧ (c|d) = (b|1) ∧ (c|d) = (bd′ ∨ bcd | 1 ∨ d) = (b(d′ ∨ cd) | 1) = b(c ∨ d′). This is reasonable since the conditional (c|d) imposes no extra constraint on b beyond (cd ∨ d′).

On the other hand, disjoining a conditional (c|d) with an unconditioned proposition b yields (cd ∨ b) not (cd ∨ d′ ∨ b). (c|d) does not make (c|d) ∨ b true whenever d is false as does the material conditional interpretation.

### 1.9 Conditional conditionals

Consider a conditional (a|b) given a second conditional (c|d). This would be ((a|b) | (c|d)). Again, in practice, we generally conjoin all conditions assumed along the way to some conclusion. This has been called the import-export rule. Here, both b and (c|d) are conditions and “a” is the qualification given those conditions.

Conditioning is commutative in most domains (e.g. most games of chance) but not in all domains (e.g. quantum measurements) but either way this can be interpreted (simplified) as (a | (b ∧ (c|d))) = (a |b(c ∨ d′)). So when a conditional (c|d) is itself a *condition* for a second conditional, it again reverts to its “material conditional” (c ∨ d′). That is,

4) ((a|b) | (c|d)) = (a | (b ∧ (c|d))) = (a |b(c ∨ d′))

This selective reversion to the material conditional has been called Gibbardian Collapse [12, 13] perhaps suggesting it poses a difficulty for this approach, but I see none.

### 1.10 Indicative conditionals

In “Indicative Conditionals” R. Stalnaker [14] provides some tests that any adequate algebra of conditionals should be able to pass:

1. Either the butler (B) or the gardener (G) did it. Therefore, if the butler didn't do it the gardener did.
2. The butler did it; therefore if he didn't, the gardener did.
3. Consider the denial of “If the butler didn't do it then the gardener did”. Does this imply that the butler did it?

If the conditional (a given b) is reduced to the “material conditional” (a or not b) then there are paradoxical results. 1. Seems quite reasonable; 2. Seems contradictory, and 3. The negation of “If the butler didn't do it then the gardener did” should not imply that the butler did it.

Let's consider these examples. 1. Can be expressed as (B ∨ G) = 1. And therefore, if B′ is given, then [(B ∨ G) |B′] = (0 ∨ G |B′) = (G|B′). The gardener did it given the butler didn't. Test 1 passed.

2. The 2^nd^ example can be expressed as ((G|B′) |B) given the butler did it, if he didn't the gardener did. But ((G|B′) |B) = (G |B′B) = (G|0), the inapplicable conditional. The conditions are inconsistent and so “no conclusion” is the result. The conditional becomes inapplicable. Test 2 passed.

Notice that had (G|B′) been rendered as the material conditional (G or not B′) = (G ∨ B) then given B, ((G ∨ B) |B) = (B|B); the contradiction disappears and “the butler did it given he did it”, which is a big problem for the material conditional rendering.

3. Denying (G |B′) - that the gardener did it given the butler didn't do it - is (G |B′)′ = (G′ |B′). It expresses the conditional statement “Given the butler didn't do it, the gardener didn't either.” There is no implication that the butler did it. Test 3 passed. [In this example, rendering (G |B′) as the material conditional (G or not B′) = (G ∨ B) seems to result in a reasonable conclusion if denied: (G ∨ B)′ = G′B′, that neither the butler nor the gardener did it.]

### 1.11 The notorious fatalism argument

R. Stalnaker^5^ [14] also relays the “notorious argument” for fatalism: I will be killed (K) or not (K′). I can take precautions (p) but they may be ineffective (q) or unnecessary (r). Thus, ((q|pK) |K) or ((r|p′K′) |K′) implies q or r --- precautions are either ineffective or unnecessary!

What's happening here is that there is a hidden assumption involving p; not all possibilities are expressed. The compound conditional is

((q|pK) |K) ∨ ((r|p′K′) |K′)

= (q|pKK) ∨ (r|p′K′K′)

= (q|pK) ∨ (r|p′K′)

= (qpK ∨ rp′K′ | pK ∨ p′K′)

But unlike (K ∨ K′) there is no assurance that (pK ∨ p′K′) will be true. If it is assumed, then (qpK ∨ rp′K′ | pK ∨ p′K′) (pK ∨ p′K′) = (qpK ∨ rp′K′), that is, either the precautions were ineffective (qpK) or unnecessary (rp′K′). So there is no conundrum here.

Suppose instead that the situation is rendered as

[K ∧ (q | pK)] ∨ [K′ ∧ (r | p′K′)]

= [K(pK)′ ∨ KqpK] ∨ [K′(p′K′)′ ∨ (K′rp′K′)]

= K(p′ ∨ K′) ∨ qpK ∨ K′(p ∨ K) ∨ rp′K′

= Kp′ ∨ qpK ∨ K′p ∨ rp′K′

= K(p′ ∨ qp) ∨ K′(p ∨ rp′)

So, either I was killed and took no precautions or they were ineffective, or I was not killed and either took precautions or they were unnecessary. These four possibilities are disjoint and have probabilities adding to 1.

### 1.12 Stalnaker Hypothesis defended

In another effort to exhibit a counter-example to the “Stalnaker Hypothesis” (the correctness of conditional probability as the most reasonable estimate of the degree of belief to attach to a conditional proposition) John L. Polluck [15] constructs what Allan Gibbard [16] refers to as an “intricately wrought example” that he says is an example of the “dispositional fallacy” rather than a true counter-example.

In case there is still some doubt about this, here is a Venn diagram that illustrates the matter. The situation is this: A certain large shipment of vases was sent, 75% ceramic (C) and 25% plastic (p). We are told that if dropped (D) a ceramic vase will always break (B), but a plastic one will never break. (Implicitly, we are to assume that the probability of a vase being dropped does not depend on whether it is ceramic or plastic.) When the vases arrive all the broken vases together with all the plastic vases were discarded (R), and of those discarded, 3/4 were plastic and 1/4 were ceramic.

Now consider (B|D), that if a vase was dropped, it broke. Prior to arrival, P(B|D) = P(B(C ∨ p) |D) = P(BC ∨ Bp |D) = P(BC|D) + P(Bp|D) = P(C|D) + 0 = P(CD)/P(D) = P(C)P(D)/P(D) = P(C) = ¾, since being dropped is independent of being ceramic. Now Polluck claims that given 75% of the discarded vases were plastic, this new information intuitively makes it unreasonable (less likely) to believe that if a vase was dropped then it broke. But he shows that since D ⊆ R (every dropped vase was discarded) therefore P((B|D) |R) = P(B | DR) = P(B|D) is still ¾.

**Figure 1:**
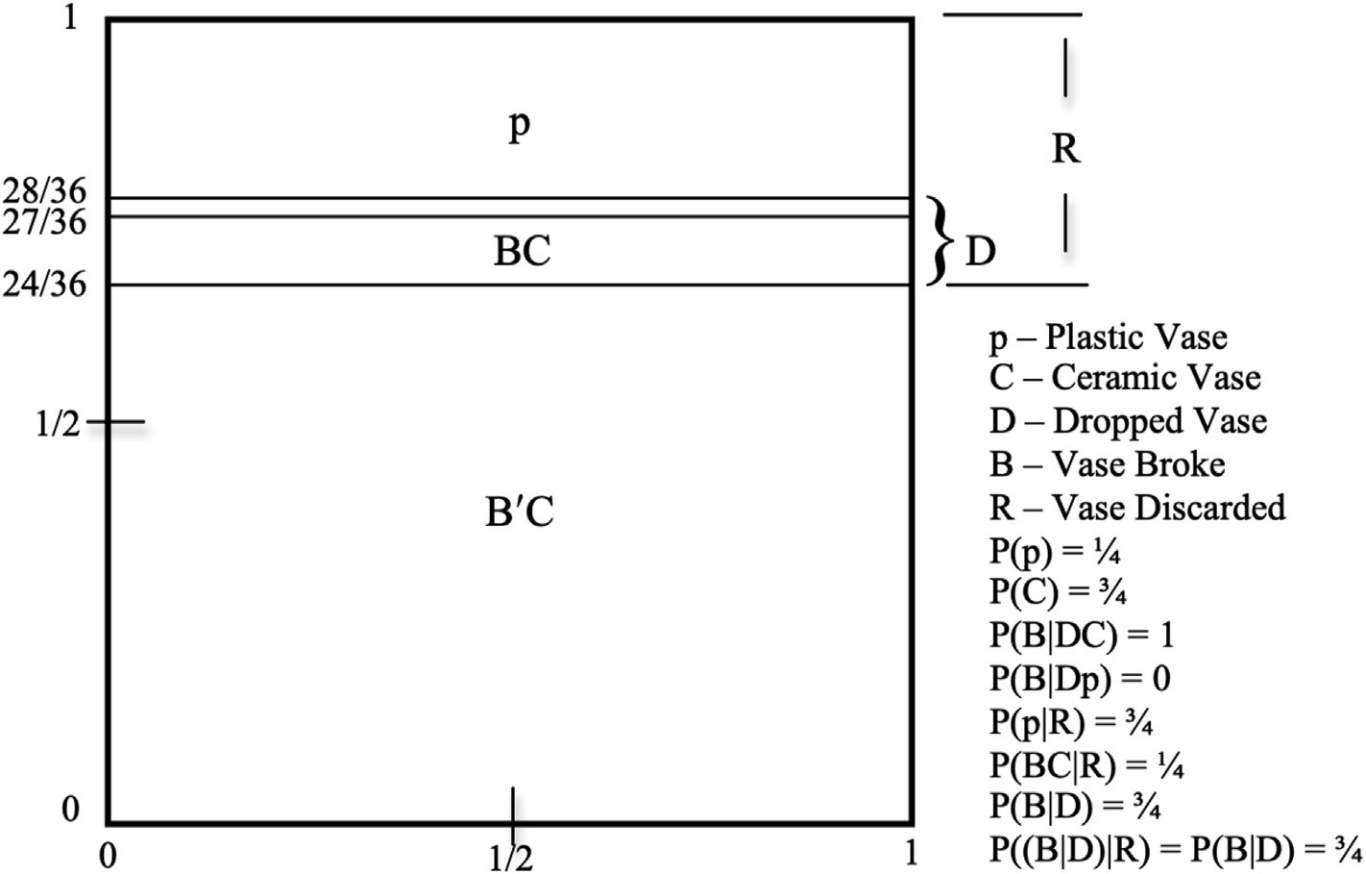
Plastic & ceramic vase delivery

But this is just another example of intuition going wrong with conditionals. Given that a vase was discarded, 3/4 is still the probability it broke if dropped. Figure 1 illustrates the matter in a Venn diagram.

### 1.13 Modus ponens

Recently, Paul Égré, Lorenzo Rossi and Jan Sprenger [17] have reexamined the adequacy of trivalent algebras with concerns about whether modus ponens can be maintained. But if (a|b) and b are conjoined, the result is ab because (a|b)b = (a|b) (b|1) = ((ab0 ∨ abb1 ∨ b1b′) | (b ∨ 1)) = (ab|1) = ab. Therefore if b is true, and a is true given b is true, then both a and b are true. This even extends to arbitrary conditionals. If (c|d) and [(a|b) given (c|d)] are conjoined, the result is (a|b) (c|d) because [(a|b) given (c|d)] = ((a|b)|(c|d)) = (a |b(c ∨ d′)), and the latter conjoined with (c|d) is (a |b(c ∨ d′)) (c|d)

= [(ab(c ∨ d′)d′ ∨ ab(c ∨ d′)cd ∨ (b(c ∨ d′))′cd | b(c ∨ d′) ∨ d]

= [(abd′ ∨ abcd ∨ (b′ ∨ c′d)cd | bc ∨ bd′ ∨ d]

= (abd′ ∨ abcd ∨ b′cd | bc ∨ b ∨ d) = (abd′ ∨ abcd ∨ b′cd |b ∨ d)

= (a|b) (c|d)

### 1.14 Tautologies

A universal (unconditioned) tautology would be of the form (a = 1). A conditional tautology would be of the form (a|b) = (1|b), which means (ab = b) and therefore that b ≤ a; b implies a; the models of b are included in the models of a. [This can also be expressed as a ∨ b′ = 1, the truth of the “material conditional”, often expressed as b ⊃ a, which notation is confusing since probabilistically, b ⊆ a when a ∨ b′ = 1. So the material conditional will always be written out as a ∨ b′. In what follows, the symbol ⊃ will not be used.]

Adding statements such as (a = 1), (a ≠ 1), (a = 0), (a ≠ 0) and (b ≤ a) to the initial Boolean algebra of propositions or events amounts to adding the existential (∃) and universal (∀) quantifiers. To say “there exists” is to assert that a certain subset is non-empty. “For all” can be expressed with ≤ or ⊆.

### 1.15 Variable universes

In some circumstances, we may need to distinguish between the probability P(q) of a proposition q versus the probability that q is a tautology. P(q) is the probability of the models in which q is true, but the probability that q is a certainty is Pr(q = 1). This happens when the universe U of possibilities can vary^6^, as for example in the game of rolling either 2 or 3 dice and considering the event of getting a sum >2. It is a certainty with 3 dice, but has just 5/6 probability rolling two dice. So if half the times, 3 dice are rolled, then (>2) is certain with probability ½. Pr[(>2) = U] = ½. Each universe may have its own internal probability function P of possibilities independent of the probability Pr of that universe among the alternate universes.

In this regard, B. Skyrms [18] employs different “propensities” that serve to alter the initial universe U or its initial probabilities in order to express counter-factual statements, which naturally shift the assumed context (universe) between what was and what would or might have been.

### 1.16 More general conditionals

My overall goal has been to provide an algebra of conditional events that joins Boolean logic and probability theory in the context of a fixed universe U of all possibilities such as in games of chance. These have been called indicative conditionals [14]. Whatever the applications of this algebra to more general problems of “counter-factual conditionals”, such as conditionals expressed in the subjunctive mood, the goal here is only to supply a missing relation “a given b” for Boolean logic [19] and for probability theory [20] by use of an “inapplicable” truth state for conditionals.

Such conditional statements as “If Oswald hadn't killed Kennedy then someone else would have” include a condition whose universe of possibilities is not clearly defined and requires interpretation. For example. this statement surely cannot be adequately rendered as “If Oswald didn't kill Kennedy someone else did”. The initial expression suggests a conspiracy as context for the conclusion. The second conditional simply expresses a logical necessity based on a conjecture.

Even more generally, modal logic includes propositions such as “p is known or believed”, “p is hoped or feared”, “p ought or should be”, and “p is good or bad”. These logics raise philosophical questions of the meaning of the contexts and conclusions and what universe they reside in. The situation gets even more complicated when two people with different knowledge say the opposite or the same thing^7^. On the other hand, temporal logics using constructions such as “p is true sometimes”, “p is true at all times”, or “p was true at one time” seem to fall nicely within the purview of probability theory and this extension algebra (B|B) of conditional events. Or do they?

### 1.17 Random variables

As mentioned before, a proposition c can be represented as a measurable indicator function defined on the set of all models, that has value 1 for a model in which c is true, and value 0 on models for which c is false. In turn a *conditional* proposition (c|d) can be represented as a *partially defined* indicator function^8^ that is 1 when cd is true, 0 when c′d is true, and undefined on models for which d is false.

Thus propositions become random variables (measurable indicator functions) on the universe of models; conditional propositions become partially defined random variables (partially defined measurable indicator functions).

These indicator functions can in turn be used as a basis for expressing conditional numerical random variables with extended operations (+, -, ÷ and |)^9^.

Along rather different lines, A. Gilio and G. Sanfilippo [11] consider an algebra of “conditional random quantities”. They employ a betting metaphor to handle the trivalent nature of conditionals: If a Boolean conditional (A|H) has conditional probability P(A|H) = x, then a player is willing to pay the amount xs (called a “prevision”) for a chance to win a monetary sum s in case (A|H) turns out to be true, 0 if (A|H) turns out to be false, and xs (the prevision is returned) in case H turn out to be false (null bet). They thus define^10^ a 3-valued random variable (A|H) = AH + xH′, which is 1 on AH, 0 on A′H and x on H′.

Unlike De Finetti's (and my) definition of (A|H) as a *partially defined* indicator function, their (A|H) is defined on the whole universe of all possible outcomes, even those for which H is false. (The collection of all possible complete assignments of values to variables is one way to specify the logical atoms or models - the possible outcomes of both systems.)

The conjunction (A|H ∧ B|K) of two of these 3-valued random quantities, A|H and B|K with conditional probabilities x and y respectively, is in general a random variable with 5 possible values including z = Min{A|H, B|K} on H′K′, that is for instances when neither H nor K is true.

In this framework, Gilio and Sanfilippo have shown^11^ that the conjunction of n+1 of these random variables p-implies (i.e. is numerically ≤) the conjunction of the first n of them. Their algebra ensures that a conjunction of conditionals p-implies its components. This preserves classical properties of Boolean logic such as monotonicity and also avoids computational complexity.

By contrast, the non-monotonic quasi-conjunction operation, although easily faithful to natural language and having clear probability applications, seemed to make calculating the implications of a set of conditionals rather difficult.

Early efforts by E. Adams [9] and later by P. Calabrese [3, 4] to use quasi-conjunction to compute all implications of a set of uncertain conditionals had run into complexity problems. The results of this paper show that this obstacle can be avoided with the right implication relation, one not defined merely by generalizing one of the equations usually used to define deduction between Boolean propositions A, B, namely A = A ∧ B, or equivalently, A ∨ B = B, or equivalently, B ∨ A′ is a certainty. In short, the quasi-conjunction of conditionals will imply each one of its components as long as the premise of that component re-conditions the quasi-conjunction. (See Section 2.).

### 1.18 The surprise execution day dilemma

It was my master's thesis advisor, Professor Karl Menger, who relayed the essence of the following rather nasty logical conundrum combining “p is known” with “time” and with the subjunctive mood: A certain political prisoner was tried and convicted of a capital crime and immediately sentenced by the judge to be executed at noon on one of the following 7 days. The judge, known for always being truthful and precise, also stipulated that at no time before the execution would the condemned man know the day of his execution until the morning of his execution day.

Later that day the convict's lawyer greatly cheered up the condemned man saying, “You can't be executed at all! Surely the judge can't have you executed on the last day because then on the night before, you'd know your execution day before the morning of the day. So clearly, the 7^th^ day can be eliminated from the possibilities. That leaves 6 days. Now having eliminated the 7^th^ day, suppose you are still alive on the night of the 5^th^ day. Then you would know ahead of time that the execution must occur on the 6^th^ day. So, the judge really can't wait until the 6^th^ day either.” Applying the same logic in turn to the 5^th^, 4^th^, 3^rd^, 2^nd^ and 1^st^ day, the lawyer concluded that the prisoner could not be executed at all since the judge always told the truth. But on the morning of the 4^th^ day the prisoner was quite surprised to learn that he would be executed at noon that day. Exactly what was wrong with the lawyer's logic? I first heard this over 50 years ago, and I'm still thinking about it.

## 2 Deduction

Let us now move on to consider deductions, which are not supposed to merely be a qualification on some context as in general are indicative conditionals. Deductions are meant to be absolute implications. No exceptions.

From the model or extensional point of view, a deductive statement between propositions such as “b implies a” means that any instance or model of proposition b is also an instance or model of proposition a. This can be conveniently represented as b ≤ a, or as b ⊆ a (inclusion). While the inclusion ⊆ is certain, the propositions themselves need not be. It follows then that P(a|b) = 1 unless b is impossible --- the empty set φ of instances, which is necessarily false. In that case (a|φ) = U, the inapplicable conditional and P(U) = is undefined. As usual, the empty event φ implies all events e. A contradiction falsely implies anything! A premise must have at least one instance to avoid this case.

### 2.1 Deduction with conditionals

The simplest (most conservative) and least imaginative extension of Boolean deduction ≤ to conditionals (a|b) & (c|d) is to define (a|b) ≤ (c|d), “(a|b) implies (c|d)”, to mean that b = d and ab ≤ cd. To say b = d is to say that the two conditionals are in the same Boolean subspace (B|b), and the inequality ab ≤ cd means that when (a|b) is true then (c|d) is true. It easily follows from {b = d and ab ≤ cd} that c′d ≤ a′b. This deductive relation, {b = d and ab ≤ cd} has been denoted ≤_Bo_ and called Extended Boolean deduction^12^.

However, suppose the condition b of the premise (a|b) properly includes the condition d of the conclusion (c|d) and still ab ≤ cd holds. Shall not that too be a valid deductive conclusion? After all, in practice, when checking the implication of (c|d) by (a|b) we would routinely restrict the condition b of the premise conditional to whatever was a condition of the conclusion (c|d).

By themselves, [without the additional conditioning of the premise (a|b) by the condition d of the conclusion (c|d)] the two inclusions {(d ≤ b), (ab ≤ cd)} define a deductive relation that has been called ≤_m∧_^13^.

It will be shown that including this additional conditioning (restricting) of the premise conditional by the condition d of the conclusion conditional (c|d) in the definition of deduction for conditionals, *makes all the difference when calculating the implications of a set of conditionals*. In fact, it greatly simplifies the treatment of deductively closed sets of conditionals as developed in P. Calabrese [2002] and [2017], which defined several deduction relations on conditionals in terms of analogous Boolean equations such as [A implies B means A ∧ B = A] or [A implies B means A ∨ B = B].

### 2.2 Optimal deductive relations ≤ for conditionals

Thus, we are led to define (a|b) *totally implies* (c|d), denoted as (a|b) ≤_T_ (c|d), in case ((a|b) |d) ≤_Bo_ (c|d).

Thus “(a|b) *wholly implies* (c|d)” means that conditioned by d, (a|b) ≤_Bo_ (c|d). Now, (a|b) conditioned by d is ((a|b) |d) = (a|bd). And (a|bd) ≤_Bo_ (c|d) means bd = d and abd ≤ cd. That is, {(d ≤ b) and (ad ≤ cd)}. Summarizing:

#### 2.2.1 Definition of total deduction ≤_T_ for conditionals

(a|b) ≤_T_ (c|d) if and only if both (d ≤ b) and (ad ≤ cd).

These two properties (d ≤ b) and (ad ≤ cd) seem to be quite serviceable for logical deduction of (c|d) by (a|b). Again, it is the implication that is certain, not necessarily either of the two conditionals. Let's check that ≤_T_ defines a deductive relation:

#### 2.2.2 Lemma: ≤_T_ is a deductive relation. That is, it is both reflexive and transitive

**Proof of Lemma 2.2.2**: Reflexivity: (a|b) ≤_T_ (a|b) since b ≤ b and abb ≤ ab.

Transitivity: Assume (a|b) ≤_T_ (c|d) and (c|d) ≤_T_ (e|f). So f ≤ d ≤ b. Therefore b includes f. Furthermore, since ad ≤ cd and cf ≤ ef, it follows that af = (af)d = (ad)f ≤ (cd)f = cfd ≤ (ef)d = ef. Therefore (a|b) ≤_T_ (e|f). So ≤_T_ is transitive.

#### 2.2.3 Properties of the deductive relation ≤_T_

From (ab ≤ cd) and (d ≤ b) it easily follows that (ad ≤ cd), but not conversely. The constraint ad ≤ cd is weaker than requiring ab ≤ cd.

Nevertheless, assuming (a|b) ≤_T_ (c|d) in this new sense, it still follows that (ab ∨ b′) ≤ (cd ∨ d′) since (ab ∨ b′) = (abd ∨ abd′ ∨ b′) and each of these 3 components is included in cd ∨ d′: abd ≤ cd, abd′ ≤ d′, and of course b′ ≤ d′.

So (a|b) ≤_T_ (c|d) implies the material conditional inequality (a ∨ b′) ≤ (c ∨ d′), which (taking complements of both sides) is equivalent to c′d ≤ a′b. Thus (a|b) ≤_T_ (c|d) also implies that when (c|d) is false then (a|b) is false. [Of course, in Boolean logic, an implication p ≤ q (p implies q) is always equivalent to its contra-positive implication q′ ≤ p′ (not-q implies not-p). That this property (modus tollens) is preserved by ≤_T_ is reassuring of its adequacy as a deductive relation for conditionals.]

However, (a|b) ≤_T_ (c|d) does not imply ab ≤ cd. Only abd ≤ cd need be true.

Note also that in general according to this deductive relation on conditionals a universally conditioned proposition or event (X|1) trivially implies a conditional (X|Y) with the same conclusion. That is, (X|1) ≤_T_ (X|Y) because Y ≤ 1 and XY ≤ XY.

The deduction (a|b) ≤_T_ (c|d) also implies that (a|b) ∧ (c|d) = (a|b) because (a|b) ∧ (c|d) = (abd′ ∨ abcd ∨ b′cd |b ∨ d) = (abd′ ∨ abcd ∨ 0 |b) since b′ ≤ d′. And since ad ≤ cd, it follows that (abd′ ∨ abcd |b) = (abd′ ∨ abd) |b) = (ab|b) = (a|b).

But (a|b) ≤_T_ (c|d) does not in general imply that (a|b) ∨ (c|d) = (c|d) unless b ≤ d, that is, unless in addition the context d of the conclusion conditional (c|d) includes the context b of the premise conditional. In that case b = d and the deduction is simply Boolean with conditionals having equivalent premises. Of course, (a|b) ≤_T_ (c|d) *does imply* that (a|d) ∨ (c|d) = (c|d).

(a|b) ≤_T_ (c|d) also obviously implies that P(a|d) ≤ P(c|d) when both are defined; it is probabilistically monotonic in this restricted sense. But P(a|b) ≤ P(c|d) may not hold.

Any other deductive relation “(a|b) implies (c|d)” for which the context b of the premise conditional (a|b) does not include the context d of the conclusion conditional (c|d) can only apply within b. Total deduction ≤_T_ is deduction in which the applicability of the premise conditional covers the applicability of the conclusion conditional.

#### 2.2.4 Definition of minimal deduction ≤_M_ for conditionals

A similar modification of the probabilistically monotonic deductive relation (a|b) ≤_pm_ (c|d) previously defined^14^ as {ab ≤ cd, c′d ≤ a′b} results in a weaker deductive relation that also has the desired simplification properties. Thus, we are led to define the “minimal” deductive relation (a|b) ≤_M_ (c|d) to mean ((a|b) |d) ≤_pm_ (c|d). This means {abd ≤ cd, c′d ≤ a′bd}.

By taking complements of the second Boolean deduction we get that (a ∨ (bd)′ ≤ c ∨ d′, i.e. a ∨ b′ ∨ d′ ≤ c ∨ d′, which is equivalent to a ∨ b′ ≤ c ∨ d′. Taking compliments again, this is equivalent to c′d ≤ a′b. So (a|b) ≤_M_ (c|d) amounts to {abd ≤ cd, c′d ≤ a′b}. As shown in the preceding subsection, these two inequalities are implied by the stronger deductive relation ≤_T_.

#### 2.2.5 Lemma: ≤_M_ is a deductive relation

**Proof of Lemma 2.2.5:** Reflexivity is trivial since (a|b) ≤_M_ (a|b) means (a|bb) ≤_pm_ (a|b), which means ab ≤ ab and a′b ≤ a′b, which clearly is true. Transitivity is not so obvious. Suppose (a|b) ≤_M_ (c|d) and that (c|d) ≤_M_ (e|f). We need to show (a|b) ≤_M_ (e|f). That is, we need {abf ≤ ef, e′f ≤ a′b}

Since (a|b) ≤_M_ (c|d), c′d ≤ a′b, and since (c|d) ≤_M_ (e|f), e′f ≤ c′d. Therefore, by Boolean deduction transitivity, e′f ≤ a′b, which proves the second Boolean deduction. We also know abd ≤ cd and cdf ≤ ef and need abf ≤ ef.

Now abf = ab(e ∨ e′)f = abef ∨ abe′f. But abe′f = 0 because e′f ≤ a′b. Therefore, abf = abef ≤ ef.

### 2.3 Deductively closed sets of boolean propositions or events

A deductively closed set (DCS) H is one for which the conjunction of any two members of H is also a member of H, and any proposition or event deductively implied (≤) by a member of H is also a member of H.

In purely Boolean logic and in the logic of probabilistic events (subsets of some universe), the set of all implications H(A) of some single proposition or event A are all the propositions or subsets X that include A. That is, H(A) = {X: A ≤ X}. Furthermore, the collection of all implications of a finite set J of Boolean propositions or events {A_i_: i = 1,2,3...n} is just the set of implications of the single proposition or event C = (A_1_A_2_A_3_...A_n_) formed by the conjunction of all members of J. H(J) = {X: C ≤ X}.

This simplicity in Boolean logic depends on the fact that the conjunction (A ∧ B) of two Boolean propositions A, B implies each of the components of that conjunction. That is, (A ∧ B) ≤ A and (A ∧ B) ≤ B. And this property easily extends to any finite collection of Boolean propositions or events. Thus, if A and B are in the set H(J) of implications of J then so is (A ∧ B) and both A and B are implied by (A ∧ B).

A deductively closed set (DCS) that is generated by a single one of its members is said to be *principle*.

### 2.4 Deductively closed sets of conditionals

Now that we have two appropriate deductive relation for conditionals with varying contexts all within a fixed universe, we can consider the implications of a collection of such conditionals.

#### 2.4.1 Theorem 1

The (principle) deductively closed set H_T_(a|b) with respect to the deductive relation ≤_T_ generated or implied by a single conditional (a|b) is {(x|y): (a|b) ≤_T_ (x|y)} = {(x|y): y ≤ b and ay ≤ xy}.

**Proof of Theorem 1:** We need H_T_ to be closed under conjunction and implication. That is, if both (c|d) and (e|f) are in H_T_, then so is (c|d) (e|f), and if (c|d) ∈ H_T_ and (c|d) ≤_T_ (e|f), then (e|f) ∈ H_T_.

H_T_ is trivially closed under implication by the transitivity of ≤_T_. So suppose both (c|d) and (e|f) are in H_T_. We want to show that their conjunction (c|d) (e|f) = (cdf′ ∨ cdef ∨ d′ef |d ∨ f) ∈ H_T_. Since d ≤ b and f ≤ b, therefore (d ∨ f) ≤ b. Secondly, since ad ≤ cd and af ≤ ef, then a(d ∨ f) = a(df′ ∨ df ∨ d′f) = (adf′ ∨ adaf ∨ ad′f) ≤ (cdf′ ∨ cdef ∨ ed′f). Therefore, (c|d) (e|f) ∈ H_T_.

#### 2.4.2 Theorem 2

If (a|b) and (c|d) are two conditional propositions or events, then (a|b) ∧ (c|d) ≤_T_ (a|b) and (a|b) ∧ (c|d) ≤_T_ (c|d). That is, the conjunction of two conditionals implies the components of that conjunction.

**Proof of Theorem 2:** By the deductive relation ≤_T_ for conditionals, {(a|b) ∧ (c|d) ≤_T_ (a|b)} means that ((a|b) ∧ (c|d) |b) ≤_Bo_ (a|b). Now (a|b) ∧ (c|d) = (abd′ ∨ abcd ∨ b′cd |b ∨ d) and conditioning by b yields ((abd′ ∨ abcd |b) = ((ab) (cd ∨ d′) |b) ≤ (ab|b) = (a|b). By symmetry, the same is true for (a|b) ∧ (c|d) ≤_T_ (c|d). So the conjunction implies both its components.

#### 2.4.3 Corollary

The deductively closed set H_T_ with respect to the deductive relation ≤_T_ generated or implied by a finite set J of conditional propositions or events is principle, being the deductive implication of the single conditional proposition formed by conjoining all members of J.

**Proof of Corollary 2.4.3:** By the theorem, the corollary is true for two conditionals. If J is a set of n conditionals (n > 2), and C is the conjunction of all members of J, then the 2^nd^ through nth conditionals of C can be conjoined to form a single conditional to which the theorem applies together with the 1^st^ conditional. Thus, the first conditional is in H_T_. Since conjunction of conditionals is commutative, the same argument can be applied to each of the other components of C. Thus C ≤ (a|b) for every (a|b) in J and C also implies the conjunction of any of the members of J and by transitivity it implies any conditional implied by members of H_T_.

The above simplification of the implications of a set of conditionals to those of a single conditional is not valid for the deductive relation ≤_m∧_. It is true of the weaker deductive relation (a|b) ≤_∧_ (c|d) defined by d ≤ b and (a ∨ b′) ≤ (c ∨ d′), but (a|b) ≤_∧_ (c|d) does not ensure that (c|d) is true when (a|b) is true. Nor does it ensure that P(a|b) ≤ P(c|d). Only by conditioning the premise conditional (a|b) by the premise d of the conclusion conditional (c|d) as part of the deductive relation does it equate to what I have denoted ≤_T_ on conditionals.

#### 2.4.4 Theorem 3

The (principle) deductively closed set H_M_(a|b) with respect to the deductive relation ≤_M_ generated or implied by a single conditional (a|b) is {(x|y): (a|b) ≤_M_ (x|y)} = {(x|y): aby ≤ xy, x′y ≤ a′b}.

**Proof of Theorem 3:** H_M_ needs to be closed under conjunction and implication. That is, if both (c|d) and (e|f) are in H_M_, then so is (c|d) (e|f), and if (c|d) ∈ H_M_ and (c|d) ≤_M_ (e|f), then (e|f) ∈ H_M_.

H_M_ is trivially closed under implication by the transitivity of ≤_M_. So suppose both (c|d) and (e|f) are in H_M_. We want to show that their conjunction (c|d) (e|f) = (cdf′ ∨ cdef ∨ d′ef |d ∨ f) = ((c ∨ d′) (e ∨ f′) | (d ∨ f)) ∈ H_M_. That is, we need ab(d ∨ f) ≤ (cdf′ ∨ cdef ∨ d′ef) and ((c ∨ d′) (e ∨ f′))′ ≤ a′b.

Since (c|d) in H_M_, {abd ≤ cd, c′d ≤ a′b}. Similarly, {abf ≤ ef, e′f ≤ a′b}. Therefore, ((c ∨ d′) (e ∨ f′))′ = (c ∨ d′)′ ∨ (e ∨ f′)′ = c′d ∨ e′f ≤ a′b ∨ a′b = a′b. That shows the second required deduction.

Next, ab(d ∨ f) = ab(df′ ∨ df ∨ fd′) = (abdf′ ∨ abdf ∨ abfd′) ≤ (cdf′ ∨ (cd) (ef) ∨ efd′), using abd ≤ cd and abf ≤ ef. That shows the first required deduction.

#### 2.4.5 Theorem 4

If (a|b) and (c|d) are two conditional propositions or events, then (a|b) ∧ (c|d) ≤_M_ (a|b) and (a|b) ∧ (c|d) ≤_M_ (c|d). That is, the conjunction of two conditionals implies the components of that conjunction.

**Proof of Theorem 4:** By the deductive relation ≤_M_ for conditionals, (a|b) ∧ (c|d) ≤_M_ (a|b) means that (abd′ ∨ abcd ∨ b′cd) (b ∨ d)b ≤ ab and a′b ≤ [(a ∨ b′) (c ∨ d′)]′(b ∨ d). Now (abd′ ∨ abcd ∨ b′cd) (b ∨ d)b = (abd′ ∨ abcd) = (ab) (cd ∨ d′) ≤ (ab), which shows the first Boolean deduction. Furthermore, [(a ∨ b′) (c ∨ d′)]′(b ∨ d) = [(a ∨ b′)′ ∨ (c ∨ d′)′](b ∨ d) = (a′b ∨ c′d) (b ∨ d) = (a′b ∨ c′d), which includes a′b. That shows the second Boolean deduction. Therefore, (a|b) ∧ (c|d) ≤_M_ (a|b). By symmetry, the same is true for (a|b) ∧ (c|d) ≤_M_ (c|d). So, the conjunction implies both its components.

#### 2.4.6 Corollary

The deductively closed set H_M_ with respect to the deductive relation ≤_M_ generated or implied by a finite set J of conditional propositions or events is principle, being the deductive implication of the single conditional proposition formed by conjoining all members of J.

**Proof of Corollary 2.4.6:** The proof is the same as the proof of the Corollary 2.4.3 for ≤_T_ with H_T_ replaced by H_M_ and ≤_T_ replaced by ≤_M_.

For simplicity, the remainder of this paper will provide examples of the deduction (a|b) ≤_T_ (c|d) as defined above in which the applicability b, of the premise conditional (a|b) covers the condition d, of the conclusion conditional (c|d). Since (a|b) ≤_T_ (c|d) implies (a|b) ≤_M_ (c|d) these examples are also examples of (a|b) ≤_M_ (c|d).

### 2.5 Examples of deduction with sets of conditionals

Suppose J = {(a|b), (c|d)} is a set of two conditionals. What are the implications with respect to the deductive relation ≤_T_ ? H(J) = {(x|y): ((a|b) (c|d) |y) ≤_Bo_ (x|y)}. These are the conditionals for which y ≤ (b ∨ d) and that satisfy (abd′ ∨ abcd ∨ b′cd) y ≤ xy.

Suppose J = {(a|b), (c|d), (e|f)} is a set of conditionals. What are the implications with respect to the deductive relation ≤_T_ ? The answer is all conditionals (x|y) for which ((a|b) (c|d) (e|f) |y) ≤_Bo_ (x|y). This means that (b ∨ d ∨ f) ≥ y and (ab ∨ b′) (cd ∨ d′) (ef ∨ f′) y ≤ xy.

#### 2.5.1 Family gathering rules

Suppose a certain dysfunctional family contains six members S = {A, B, C, D, E, F} and they only gather according to the following rules:

- If A is present then so are B and C present. That is, (BC | A).
- If D or E is present then A is not present. That is, (A′ |D ∨ E)
- If F is present then C is too. That is, (C |F).

What are the possible gatherings of this family? (Obviously, this example can be interpreted as a set of conditional inclusion relationships between subsets.)

The quasi-conjunction K of the 3 conditionals is

(BC | A) (A′ |D ∨ E) (C | F)

= [(BC ∨ A′) (A′ ∨ (D ∨ E)′) (C ∨ F′) | (A ∨ (D ∨ E) ∨ F)]

and the conclusion of this conditional can be simplified as:

= (BC ∨ A′) (A′ ∨ D′E′) (C ∨ F′)

= (A′ ∨ (BC) (D′E′)) (C ∨ F′)

= A′(C ∨ F′) ∨ (BC) (D′E′) (C ∨ F′)

= A′C ∨ A′F′ ∨ BCD′E′

= BCD′E′ ∨ A′(C ∨ F′)

= ABCD′E′ ∨ A′(C ∨ F′)

So K = (ABCD′E′ ∨ A′(C ∨ F′) | (A ∨ D ∨ E ∨ F))

Let's check this: Conditioning K by A reduces K to (ABCD′E′ | A), which preserves the rules regarding A. Notice that F may be present or not. The rules are neutral about F given A.

Conditioning K by D reduces K to (A′(C ∨ F′)D | D), events for which A will be absent, D present, and either C present or F absent. Note that B, or E, or both may or may not be present at the gathering given D is present. Conditioning K by either B or E along with D simply adds them to the gathering without any further implications.

Conditioning K by E reduces K to (A′(C ∨ F′)E | E). These are gatherings without A, with E, and with either C or not F.

Conditioning K by F reduces K to (BCD′E′F ∨ A′(CF) | F) = ((CF) (BD′E′ ∨ A′) | F) = ((CF) (ABD′E′ ∨ A′) | F) = (A′CF ∨ ABCD′E′F | F) = (A′CF|F) ∨ (ABCD′E′F | F). In such gatherings given F is present, either A is present along with B, C but not D or E, or else A is not present, C is present, and B, and D may or not be present.

Conditioning K by C reduces K to

(ABCD′E′ ∨ A′(C ∨ F′) | (A ∨ D ∨ E ∨ F) C)

= (ABCD′E′ ∨ A′C | (A ∨ D ∨ E ∨ F) C)

But (A ∨ D ∨ E ∨ F) does not in general include C. So, the rules do not in general imply anything more given C is present.

Similarly, since (A ∨ D ∨ E ∨ F) ≥ B is not guaranteed, given B is present the three rules embodied in K do not in general imply anything more.

#### 2.5.2 The absent-minded coffee drinker^15^ (revisited)

So far, we have considered only so-called “indicative conditionals”, for which the two components are members of a sample space of probabilistic events or equivalently, a set of logical propositions whose models (worlds) in which they are true form such a sample space. But some conditionals incorporate propositions that are hard to express indicatively. Consider the following example.

“Since my spoon is dry (D) I must not have sugared (G) my coffee, because the spoon would be wet (D′) if I had stirred (S) the coffee, and I wouldn't have stirred it unless I had put sugar in it.”

The above coffee drinker seeks to deduce whether his or her coffee is sugared (G) based on the *observation* that the spoon is dry (D) together with the two conditional statements, one of which is (D′|S) that the spoon wouldn't be dry if the coffee was stirred. I am interpreting the second conditional - “I wouldn't have stirred it unless I had put sugar in it” - as (S′|G′) whose contrapositive (2-valued logical equivalent) is (G|S) that “if I stirred my coffee, then I must have sugared it”.

This 2^nd^ conditional statement might be interpreted to mean the conjunction of (S′|G′) and (S|G), the latter being “if I sugared my coffee, I would have stirred it”. But let's leave that interpretation out for the time being.

We form K

= (D) (D′|S) (S′|G′) = [(D|1) (D′|S)] (S′|G′)

= (D1S′ ∨ D1D′S ∨ 1′D′S | 1 ∨ S) (S′ | G′)

= (DS′ ∨ 0 ∨ 0 | 1) (S′ | G′)

= (DS′|1) (S′|G′)

= [(DS′G ∨ DS′S′G′ ∨ 1′S′G′) | (1 ∨ G′)]

= (DS′G ∨ DS′G′ ∨ 0 | 1)

= (DS′| 1) = DS′

Had (S′|G′) been replaced by its contra-positive (G|S) the result would be the same with an easier computation:

(D) (D′|S) (G|S) = (D) (D′G|S) = (DS′ ∨ 0 ∨ 0 | 1) = (DS′|1) = DS′

So with no further conditions the spoon is dry and the coffee is not stirred, but there is no implication that the coffee is or is not sugared (G′). Based on the conditionals as expressed, the coffee might still be sugared and not stirred; (S|G) is not included in the initial interpretation. Stirring the coffee given it has been sugared is not part of the initial interpretation.

If the conditional (S|G) is included with the other three conditionals, then K = (DS′|1) (S|G) = (DS′G′ ∨ 0 ∨ 0 | 1 ∨ G) = (DS′G′ | 1) = DS′G′. So the coffee drinker could conclude that the coffee was not yet sugared, nor stirred.

#### 2.5.3 The penguin problem^16^ (revisited)

In the above deduction examples, the conditionals were all taken to be totally true. However, conjoining partially true information with totally true information degrades or obscures the absolutely true information while it may improve the probability of the partially true information.

Consider the following conditional statements: “Birds fly (F)”, “Penguins (p) are birds (B)” and “Penguins don't fly.” These can be expressed as (F|B), (B|p) and (F′|p). What are the implications of these three conditionals?

Notice that (F|B) is not totally true; it might be expressed as the conditional probability P(F|B) >> 0. On the other hand, (B|p) is totally true by definition of a penguin; p ≤ B. So (B|p) = (Bp|p) = (p|p) = (1|p) and P(B|p) = 1. A bird is certain, given a penguin. The conditional (F′|p) that penguins don't fly is probably an empirical observation but not part of the definition of a penguin. Thus, it might be expressed as P(F′|p) ∼ 1. Or for anatomical reasons perhaps penguins can't fly; p ≤ F′. In that case p ≤ B and p ≤ F′ conjoin as p ≤ F′B.

In either case, since their conditions are equal, (B|p) (F′|p) can be expressed as (BF′|p), penguins are birds that don't fly.

Conjoining (F|B) with (BF′|p) yields K = (FBp′ ∨ 0 ∨ 0 |p ∨ B) = (FBp′ |B) = (Fp′|B).

Now given a bird (B), K is unchanged: ((Fp′ |B) |B) = (Fp′|B). Given a bird the qualification is a flying non-penguin. But given a penguin p, K becomes (K|p) = (FBp′p |p) = (0|p), a contradiction. If (all) birds fly and penguins are birds then logically, penguins must fly or else there is a contradiction.

However, conjoining partially true conditionals such as (F|B) with totally true ones always degrades the available information. In this case, it seems to lose the information that penguins are non-flying birds.

What is needed is a probability analysis of the partially true conditional (F|B) that also incorporates the totally true information.

Since (F|B) is definitely not a certainty, we can start with one, namely, (F ∨ F′ |B) and conjoin the other certain information, (BF′|p), with it. This yields:

K = (F ∨ F′ |B) (BF′|p)

= [(F ∨ F′)Bp′ ∨ (F ∨ F′)B(BF′p) ∨ B′(BF′p) | (B ∨ p)]

= [FBp′ ∨ F′Bp′ ∨ (0 ∨ F′Bp) ∨ 0 | B]

= (FBp′ ∨ F′Bp′ ∨ F′Bp | B)

= (Fp′ ∨ F′p′ ∨ F′p | B)

Given a penguin p, K becomes (K|p) = ((Fp′ ∨ F′p′ ∨ F′p | Bp) = (F′B|p). That is, given a penguin you get a non-flying bird. Furthermore, P(F′B|p) ∼ 1.

Given a bird B, K is unchanged, (Fp′ ∨ F′p′ ∨ F′p | B) = (p′ ∨ F′p | B). So given a bird, you get either a non-penguin bird (that may or may not fly) or a non-flying penguin, and P((Fp′ ∨ F′p′ ∨ F′p | B) = 1.

The conditional (p′ ∨ F′p | B) can be expressed as (p′ ∨ F′ | B) = ((pF)′|B). Given a bird, you never get a flying penguin. P((pF)′| B) = 1.

Furthermore, since Fp′, F′p′ and F′p are disjoint, P(Fp′ ∨ F′p′ ∨ F′p |B) = P(Fp′|B) + P(F′p′|B) + P(F′p|B) = 1.

This formula allows for non-penguin, non-flying birds like ostriches.

### 2.6 Computer calculations

When it comes to performing *practical* logical and probabilistic calculations starting with a finite set of known or assumed propositions and conditional propositions (all variables having a finite number of possible values), the services of a computerized expert system are essential.

In principle, the universe U of all possible value assignments of n variables, each with m possible values, has m^n^ members called atoms. And the set 2^U^ = {all subsets of these atoms} constitutes the universe of all potential events (propositions) that can be formed with the initial variables. 2^U^ has 2 raised to the power m^n^ members. Even for n = m = 4, this is 2^64^, which is more than 18,446,700,000,000,000,000 members. That's over 18,400 trillion, a number that make even the US national debt of $28 + trillion look small! And this results in 3^64^ different possible *conditional* propositions --- “rules” in computer jargon.

The four operations 1) – 4) faithfully simplify compound and nested conditionals. Theorem 2 simplifies the calculation of the deductive implications of a pair of conditionals, and its corollary extends this to any finite set of conditionals. The set of implications of a set J of conditionals with respect to the new deductive relations ≤_T_ and ≤_M_ is shown to be the implications of the single conditional formed by the conjunction of the members of J.

While these operations and theorems can help with the complexities of information processing, lack of sufficient facts or initial assumptions requires methods to give equal weight to otherwise unconstrained variable assignments. For this complexity problem, the concept of information entropy has come to the rescue of otherwise intractable calculations. For example, the expert system SPIRIT developed at FernUniversität Hagen, Germany [23] employs these and graph theory methods to link clusters of related variables and by assuming conditional independence to fill in any information gaps, efficiently finds the optimal (maximum information entropy) distribution given the initial assumptions.

## 3 Summary & conclusions

Section 1 provides a general introduction to this algebra of conditionals and addresses various tests, questions, examples and objections raised about the efficacy of this algebraic extension of Boolean logic and conditional probability.

Section 2 of this paper defines two new, simplifying deductive relations ≤_T_ and ≤_M_ between uncertain conditional propositions (a|b) and (c|d). One is (a|b) ≤_T_ (c|d), which means {d ≤ b, ad ≤ cd}. The other is (a|b) ≤_M_ (c|d), which means {c′d ≤ a′b, ad ≤ cd}. ≤_T_ implies ≤_M_. With respect to each of these deductive relations, it is proved that the implications of a finite set of conditionals are also the implications of the single conditional formed by conjoining all of them with the so-called quasi-conjunction of two or more conditionals. These new deductive relations avoid the complexity problems previously associated with deduction with the quasi-conditional operation. The quasi-conjunction of a finite set S of conditionals encodes their conjoined constraints given the disjunction of the premises of the conditionals of S, but the individual premise x of an arbitrary conditional (y|x) in S must condition that quasi-conjunction in order to imply y. The quasi-conjunction of all members of S implies any member (y|x) of S when conditioned by x.

## Declarations

### Author contribution statement

Philip Calabrese: Conceived and designed the analysis; Analyzed and interpreted the data; Contributed analysis tools or data; Wrote the paper.

### Funding statement

This research did not receive any specific grant from funding agencies in the public, commercial, or not-for-profit sectors.

### Data availability statement

No data was used for the research described in the article.

### Declaration of interests statement

The authors declare no conflict of interest.

### Additional information

No additional information is available for this paper.

## Footnotes

1 See [4] and Chapter 6 of [3].

2 See [5], p. 234.

3 Unknown to me at the time, Bruno de Finetti [8] early gave this definition of equivalence for conditional statements. Also see [5], Definition 3.5.4, p 209.

4 Earnest Adams [9] called 1) & 2) *quasi-operations*. They were rediscovered by the author and expressed as axioms in [5], p 216.

5 P. 204.

6 See Section 4.5, “The Probability of Entailment”, p. 220 of [3].

7 See p. 232 of [13].

8 See for example Representation as Measurable Propositional Functions, Section 5.8 (234-5) of [5, 10] or Chap. 4 (74–92) of [3].

9 See “Operating on Functions with Variable Domains”, P. Calabrese [10].

10 [11], p. 82.

11 [11], Theorem 7, p.84.

12 See p. 163 of [4].

13 See p. 165 of [4].

14 See p. 165 of [4].

15 See [21], p. 27, #78.

16 See J. Pearl [22].
